# The Insulin Regulatory Network in Adult Hippocampus and Pancreatic Endocrine System

**DOI:** 10.1155/2012/959737

**Published:** 2012-09-04

**Authors:** Masanao Machida, Shin Fujimaki, Ryo Hidaka, Makoto Asashima, Tomoko Kuwabara

**Affiliations:** Research Center for Stem Cell Engineering, National Institute of Advanced Industrial Science and Technology (AIST), Central 4, 1-1-4 Higashi, Tsukuba Science City 305-8562, Japan

## Abstract

There is a very strong correlation between the insulin-mediated regulatory system of the central nervous system and the pancreatic endocrine system. There are many examples of the same transcriptional factors being expressed in both regions in their embryonic development stages. Hormonal signals from the pancreatic islets influence the regulation of energy homeostasis by the brain, and the brain in turn influences the secretions of the islets. Diabetes induces neuronal death in different regions of the brain especially hippocampus, causes alterations on the neuronal circuits and therefore impairs learning and memory, for which the hippocampus is responsible. The hippocampus is a region of the brain where steady neurogenesis continues throughout life. Adult neurogenesis from undifferentiated neural stem cells is greatly decreased in diabetic patients, and as a result their learning and memory functions decline. Might it be possible to reactivate stem cells whose functions have deteriorated and that are present in the tissues in which the lesions occur in diabetes, a lifestyle disease, which plagues modern humans and develops as a result of the behavior of insulin-related factor? In this paper we summarize research in regard to these matters based on examples in recent years.

## 1. Introduction

Insulin is a hormone that helps cells store sugars and fats as energy sources, and a variety of problems develop in the circulatory organs including heart when its synthesis and secretion become impaired [1–4]. The fact that insulin is also essential to maintaining brain function is important [5–8], and in addition to the retinopathy and peripheral neuropathy that are known complications of diabetes, changes in insulin signals in the brain of diabetic patients have a pronounced impact on neuropathy in the central nervous system (CNS), especially in the hippocampus [9]. Actually, not only are diabetic patients at increased risk of contracting neurodegenerative diseases and psychiatric disorders such as Alzheimer's disease, Parkinson's disease, depression, and Huntington's disease, but diabetes has also been experimentally shown to have a great effect on the functions of the neural circuits in the hippocampal region [9–13].

The hormone insulin is secreted by the *β* cells that compose the pancreatic endocrine tissue in response to the environment and conditions in which the individual and the organs and tissues find themselves, and its metabolic mechanisms are strictly regulated [1–6]. Insulin also plays a major role in the stage that controls differentiation, especially by tissue stem cells, into almost all of the cells that compose the organ or the body. Under conditions in which the amount of insulin levels is reduced, the proliferative and maintenance functions of undifferentiated stem cells themselves (self-renewal capacity) are often consistently suppressed, even in different lineages. On the other hand, based on research reports in recent years it is now clear that insulin-activated signal transduction mechanisms play a major role in regulating their differentiation pathways at the stem cell differentiation induction stage in the various tissues [5–9].

## 2. Neural Stem Cells and Insulin

Stem cells retain pluripotency, which is the source of their ability to differentiate into various cell types that compose tissues and to fulfill their functions. When organs are formed during embryonic stage, organogenesis is achieved by vigorous renewal by embryonic stem cells, which have very high proliferative capacity, and by orderly differentiation of tissue cell groups for the patterning. Stem cells can renew themselves almost indefinitely and differentiate into the cells that compose tissues. Asymmetric cell division allows stem cells to self-renew and produce another cell that undergoes differentiation. Stem cells are broadly classified into two types: stem cells in the embryo stage and adult stem cells, which are present in the tissues of adults. Neural stem cells, which are responsible for neurogenesis, are present not only during embryonic and perinatal stages but also in the adulthood stage. 

**Figure 1:**
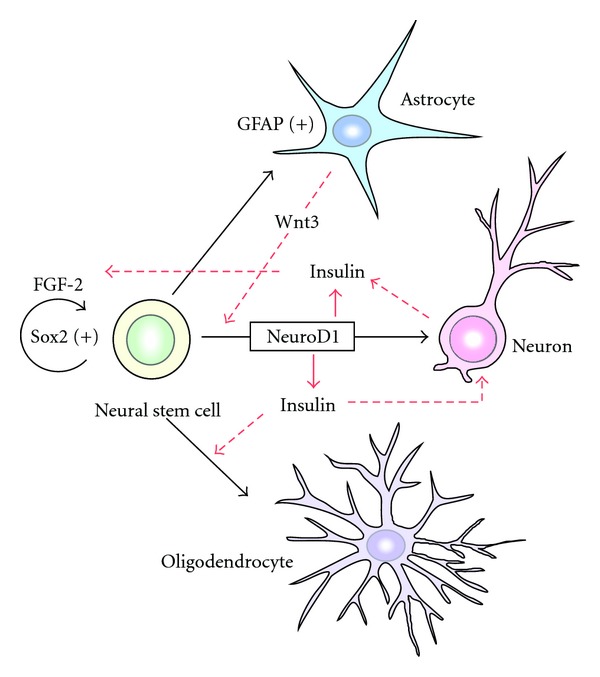
Schematic representation of the signals and transcription factors regulating adult neurogenesis. Undifferentiated adult neural stem cells express Sox2 transcription factor for the self-renewal function. FGF-2 promotes the proliferation of neural stem cells and insulin and IGF-1, and IGF-2 support the process. Astrocyte-secreted Wnt3 promotes the neuronal differentiation from neural stem cells by the activation of NeuroD1 transcription factor in the neuronal progenitor cell. The NeuroD1 transcription factor triggers the expression of insulin gene. Insulin, IGF-1, and IGF-2 promote the oligodendrocyte differentiation from neural stem cells. They also promote neuronal survival and possess the protection ability of mature neurons by preventing their natural cell death.

In recent years, it has been shown that neural stem cells are present in adult mammalian brain. They self-renew by continually dividing, as well as differentiate into functional neurons and glial cells (astrocytes, oligodendrocytes) (Figure 1). The property of neural stem cells, such as self-renewal and the multipotency, is finely modulated by both cell-intrinsic and cell-extrinsic factors. It is well known that dietary restriction and exercise (mild running, etc.) enhance adult neurogenesis. On the other hand, severe stresses, brain inflammation, and the aging impair the rate of neurogenesis significantly. High-fat diets and obesity pose serious health problems as well as the decline of adult neurogenesis. To control the coordinate energy intake and expenditure, insulin signaling is utilized. Insulin is an important neuromodulator, contributing to neurobiological processes, in particular energy homeostasis and the cognitive function. Adult neural stem cells sense and respond to changes in energy homeostasis occurring locally in the brain and systemically in the mammalian organism. The regulatory system from an undifferentiated neural stem cell to a differentiated neuron, astrocyte, or oligodendrocyte is associated with various transcriptional changes, including numerous genes associated with metabolism and energy sensing such as insulin signal transduction pathways and insulin receptors (Figure 1). Understanding the hormonal aspects of how adult neurogenesis is regulated may lead new strategies for treating neurodegenerative disorders.

## 3. Diabetes and Hippocampal Neurons

As a result of high-calorie diets and sedentary lifestyles, diabetes is rapidly becoming more prevalent and appears to negatively impact the brain, increasing the risk of depression and dementia. The diabetes patients of type-1 (caused by insulin deficiency) or type-2 (mediated by insulin resistance) exhibit impaired cognitive function compared to age [14]. The incidence of Alzheimer's disease is almost twice that in a nondiabetic population [10]. Diabetes-related cognitive dysfunction has been controversial as to its underlying cause, whether it is the result of both microvascular and macrovascular cerebral diseases, hypoglycemic episodes with subsequent neuronal loss, the direct neuronal damage caused by chronically elevated intracellular glucose concentration in hyperglycemia, or the decline of neurogenesis. Recent studies suggest that diabetes produces a variety of neurochemical, neuroanatomical and behavioral changes that are indicative of accelerated brain aging in the hippocampus, including morphological changes [15], accumulation of oxidative stress markers [16], electrophysiological changes [17], neuroendocrine changes [18], and cellular molecular changes [19]. Diabetes rats exhibit dendritic atrophy of hippocampal pyramidal neurons, decreases in spine density, synaptic reorganization in the dentate gyrus (DG), increased neuronal vulnerability and reductions in cell proliferation and neurogenesis [20–22]. Since insulin replacement inhibits or reverses these neurological deficits in diabetes animals [23, 24], these results support the emerging and expanding hypotheses regarding the important role of insulin in the CNS, especially for hippocampal neurons. 

## 4. *NeuroD1* Gene for the Insulin Expression in Both Pancreas and Hippocampus

As an example of control in the hippocampal nervous system in the brain, insulin increases the tolerance of mature neurons to toxicity and has a protective function that keeps the network functions of the neurons in an active state [25, 26] (Figure 1). Insulin also exercises control of the fate-determining mechanism (cell fate choice) of adult neural stem cells, that is, it newly promotes induction of undifferentiated neural stem cells to differentiate into oligodendrocytes [27], which have the function of protecting neurons. Moreover, insulin promotes the function of fibroblast growth factor 2 (FGF2), which has an important role in maintaining neural stem cells in the undifferentiated state, and it also plays a major role in the stem cell self-renewal stage, that is, it strongly activates stem cell proliferation [28–32].

The hippocampus is one of the parts of the brain in which severe damage occurs in both diabetic patients and patients with neurological diseases, including Alzheimer's disease [9–13]. The hippocampus governs human memory and learning functions, and all throughout life new neurons are generated daily by the neural stem cells located in the DG region, which is the region of the hippocampus where adult neurogenesis occurs [33–39]. The newly generated neurons construct new neural circuits with existing neurons, and a variety of extracellular factors, intracerebral neurohormones, and neurotransmitters whose function is to transmit information between neurons are synthesized and secreted, and they support the functions of the neural circuits. Not only a decrease in the neural activity of information transmission in these neurons but also a decrease in the phenomenon of neurogenesis by neural stem cells occurs due to the insulin deficiency in diabetic patients, and as a result there is a large decline in learning and memory capacity, which is governed by the hippocampus [5–13].

Research on the molecular mechanisms that control neurogenesis by the adult neural stem cells in the hippocampus has been vigorously pursued [36, 40, 41]. The paracrine factor Wnt3 is synthesized by astrocytes [42–44], which are a neurogenesis niche environment in the hippocampus. Neuronal differentiation is initiated when the *NeuroD1 *gene is activated by the Wnt3 factor produced by the glia [42–44]. NeuroD1 is also a transcriptional factor that directly activates the insulin gene [45–47]. *NeuroD1*-gene-deficient mice lack the DG region, which is the site of neurogenesis in the hippocampus, and that causes a fatal functional impairment of the nervous system [47, 48]. NeuroD1-deficiency in mice causes severe diabetes and perinatal lethality because NeuroD1 is required for insulin gene expression [45, 46]. Rubio-Cabezas et al. investigated human subjects and sequenced the *NeuroD1 *gene in 44 unrelated patients with permanent neonatal diabetes, result in that homozygous mutations in *NeuroD1* were identified in diabetes patients [49]. The study shows that diabetes, neurological abnormalities including cerebellar hypoplasia, learning difficulties, sensorineural deafness and visual impairment result from the loss of function mutations in *NeuroD1*. These reports indicate the critical role of NeuroD1 in both the endocrine pancreas and the CNS not only in animal models (rodents) but also in humans.

## 5. Insulin Expressions in CNS

Neuronal synthesis of insulin has been debated for long time. *De novo* insulin synthesis in mammalian brain has been supported by the detection of preproinsulin I and II mRNA in rat fetal brain and cultured neurons and also by insulin immunoreactivity in neuronal endoplasmic reticulum, axon, dendrites, and synapses [50–56]. Singh et al. reported that insulin is expressed in cultured rat hippocampal neurons but is not expressed in peripheral sympathetic neuronal cells [57]. Devaskar et al. reported that rabbit hippocampus expressed insulin mRNA apparently by using RNase protection assays and in situ hybridization analysis [58]. High performance liquid chromatography, radioimmunoassay, and [35S]cysteine metabolic labeling of cultured neuronal and glial cells indicated extracellular secretion of immunoprecipitable insulin by neurons only [58]. Further studies provided clear evidence that insulin synthesis occurs in mammalian brain CNS, where it may reach high levels [57, 59–61]. Consistent with these studies, we also reported that *de novo* insulin synthesis was performed in adult neural stem cell culture *in vitro* and in newborn neurons in the rat and mouse hippocampus *in vivo *[62]. 

Importantly, the mentioned NeuroD1 transcriptional factor is essential for expression of target insulin gene in CNS. Wnt3 stimulates their activation as stem cell niches-secreting paracrine factor in hippocampal DG [42–44]. The localization of insulin-expressing neurons involves olfactory bulb and higher order association which are rich in dopaminergic norepinephrinergic innervation [57, 62]. These same areas express insulin receptors [63], suggesting autocrine or paracrine action of the insulin in adult hippocampus. Since hippocampal neurogenesis is coupled with expression of NeuroD1 [43, 46–48, 64], the expression (and the secretion) of insulin in newborn neurons via the NeuroD1 transcription factor may be upregulated in a treatment that promotes adult neurogenesis in diabetes patients.

## 6. Regenerative Therapy for Diabetes Using Adult Neural Stem Cells

**Figure 2:**
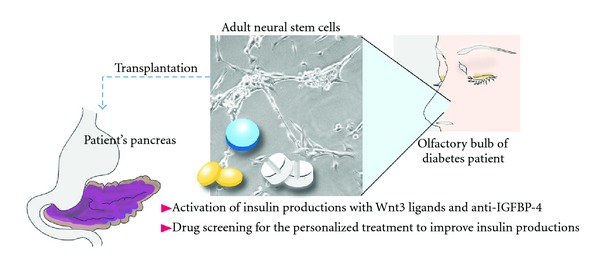
Concept of regenerative therapy for diabetes using autologous neural stem cells from the olfactory bulb. Adult neural stem cells are extracted form the olfactory bulb surgically using an endoscope. Since neural stem cells in diabetic animals had been found to contain higher IGFBP-4 (the Wnt inhibitor) and lower levels of Wnt3 (activators for insulin production via the NeuroD activation) than wild-type animals, treating the cultured neural stem cells with Wnt3 ligands and anti-IGFBP-4 (neutralizing antibody against the IGFBP-4 protein) rescues insulin expressions during *ex vivo* culture on the collagen sheets. This step would be improved by the various types of drug screening and/or also for order-made cell screening system for the personalized therapy.

Transplantation of pancreatic islets (islets of Langerhans) from the pancreas of a different donor is an effective treatment method for type-1 diabetes. However, the problem of shortage of human donors for pancreatic islet transplantation is extremely serious. As described previously, adult neurons existing in our brain neurogenic area, such as hippocampus, have the intrinsic capacity to produce insulin, implying that adult neural stem cells have a potential as a cell source for the stem cell-based transplantation therapy of diabetes (Figure 2). Adult neural stem cells can be established and cultured by collection from the olfactory bulb [65–68], which is easier location to collect multipotent neural stem cells than the hippocampus. It has been confirmed that when adult neural stem cells are collected from diabetic rats, established as cultures, and transplanted into the pancreases of diabetic rats once a state of ready insulin production has been achieved, a decreased blood glucose level can be maintained [62]. In addition, when the transplanted neural stem cells were removed from the alleviated diabetic rats, the blood glucose level again increased [62]. Since the strategy is based on autologous transplantation of cells (adult stem cells from patient's olfactory bulb: endoscopic collection of neural stem cells from the olfactory bulb is preferable to collection of intracerebral neural stem cells by means of difficult surgery), there are no donor issues and no concerns about the adverse effects of immunosuppressive agents. The insulin-producing cells are continuously replenished by adult neural stem cells and therapeutic efficacy is thus maintained. Another advantage is that the treatment has a low carcinogenesis risk and is considerably safe because the procedure involves no gene transfer. 

However, to make it closer to reality of the treatment using adult neural stem cells for diabetes, there are several challenges and issues to be overcome. It is important to evaluate the method using adult neural stem cells derived from large model animals more close to human, such as monkeys and pigs. During the time-period that adult neural stem cells are extracted from the CNS, such as olfactory bulb, and expanded to transplant to the pancreas of the patients, the improvement of the technology and methodology to activate the neural stem cells with high quality to express the insulin is also required. Increasing the insulin expression capability by *ex vivo* culture with recombinant Wnt3 protein (activator of NeuroD1 expression) and antibody against IGFBP-4 (inhibitor of Wnt signaling) is essential for the scheme that was examined in rodent study [62] (Figure 2). This step can be further improved by the various types of drug screening and/or also for order-made cell screening system for the personalized therapy. The screening system usines adult neural stem cells that assume drug discovery for diabetes patients with neurological diseases is considered to be available, in conjunction with a pharmaceutical company with chemical compound libraries. If declined ability of the CNS and the neural stem cell function could be improved by the medication, the synergistic effect of diabetes treatment with combined transplantation therapy would be expected. 

In terms of stem cell-based regenerative medicine, it is more elegant to activate the adult stem cells present in the patient's body safely, and in a way that is closer to that which occurs naturally without stressful surgeries in future. Monitoring of patient's insulin production ability on the olfactory bulb-derived adult neural stem cells would be also useful in both diabetes treatments and neurological diseases caused by low-insulin levels.

## 7. Similarity between Adult CNS and Pancreatic Endocrine System

A basic mechanism of expression of a certain absolutely essential biological factor, insulin, that has correlative similarities has been demonstrated in very different tissues that are far apart from each other in the body: the pancreas and the hippocampus. Because the nervous system is the source of insulin production in invertebrates, such as the fly [69, 70], it is very interesting that in maintaining the function of both the CNS and the pancreatic endocrine system the insulin regulatory system plays a major role in the mechanism that controls stem cell differentiation in both tissues and has been preserved during evolution. Comparisons and analyses of the functions of neural circuits in the CNS and hormone metabolic system in the pancreatic endocrine system are expected to provide new clues to understanding and analyses based on new approaches in the field of research on the control mechanisms of neuronal circuits. For example, is not there a possibility that the insulin control network, that is, proliferation and differentiation of stem cells and protection of neurons in the CNS (Figure 1) functions similarly in the cells that build up pancreatic endocrine tissue (*α* cells, *β* cells, *δ* cells, and *γ* cells)? 

Actually, niche environment correlations are observed. An astrocyte marker in CNS, GFAP, is expressed in a similar manner in the *α* cells of the pancreas together with glucagon [61, 71, 72]. Another astrocytic gene, S100beta, is also expressed in pancreatic GFAP-positive cells [73]. Pancreatic *δ* cells may be regulated under gene control in the similar way in part as interneurons at hilus region in the hippocampus, since they share same expression somatostatin as typical marker of the cell population [74]. Somatostatin is a multifunctional peptide with variety of functions, and it has been associated with disease progression, such as neurological disorders including Alzheimer's disease and multiple endocrine neoplasia [75, 76]. The understanding of the regulatory system of differentiation of each lineage from adult neural stem cells may contribute to develop new tools of the therapy of diseases affecting both pancreatic endocrine system and CNS.

## 8. Sensor Cell Populations That Control the Cell Fate Choice of the Stem Cells-Wnt3 and Insulin

Astrocytes function as the neural stem cell niche in the hippocampus, and they not only have important functions that maintain neuron functions and support neural activity, but also support neurogenesis by undifferentiated adult neural stem cells. One of its molecular mechanisms consists of secretion of Wnt3/Wnt3a protein, which is important for triggering neurogenesis (activation of *NeuroD1 *gene) and controlling stem cell behavior. Glycogen synthase kinase 3 (GSK3) is a ubiquitously expressed serine/threonine kinase that is central to insulin and Wnt signaling [77]. In insulin-signaling, GSK3 constitutively inhibits glycogen synthesis by the phosphorylation. In the canonical Wnt signaling that is activated by Wnt3/Wnt3a, beta-catenin is the substrate for the GSK3. Wnt and insulin both negatively regulate GSK3, albeit through two disparate signaling cascades [78]. Insulin target genes contain insulin response elements, which bind a variety of transcription factors [79]. Insulin receptors are abundant in neurons in cell bodies and synapses and less abundant in glia [80–84]. Wnts can signal through several different types of receptors, but the most widely recognized Wnt receptors are Frizzled proteins (Fzd). Fzd receptor subtypes and related signaling molecules are expressed in the adult hippocampus, where they play roles in the survival, function, and plasticity of neurons. In mice lacking Wnt3a, the hippocampal formation is disrupted, suggesting Wnt3a-medated signaling is crucial for the normal growth of the hippocampus [85].

Astrocytes have been found to retain sensor function that transmits the environmental signals to the stem cells, by producing the paracrine factor Wnt3 in response to the circumstances [42–44]. The frequency of neurogenesis declines with age, and it varies with the environment in which individuals find themselves, such as stress or disease, suggesting that neurogenesis in the hippocampus is regulated by molecular mechanisms that are capable of readily changing in response to external stimuli [44, 86]. Some “external stimuli,” for example, exercise and an enriched environment, increase the formation of the network of new neurons in the hippocampus, while others, for example, stress, disease, and aging, reduce it, and the patterns of expression of large numbers of gene groups change in a variety of ways.

## 9. Developing New Strategy to Treat Diabetes and Neurological Diseases

When we observe the homology between the pancreas and hippocampus by comparing them against each other, very interesting questions arose as to whether there might also be a cell population that has a sensor function in the pancreatic endocrine system. Hardly anything has yet been learned about the behavior according to the stage of disease progression or biological information, such as age, of the *α* cells in pancreatic endocrine tissue, which correspond to the Wnt3 factor producing ability of the hippocampal astrocytes, and future research is being awaited. If it were possible to control the Wnt3 producing ability of hippocampal astrocytes and pancreatic *α* cell niches, it might lead to improvement of the tissue stem cells whose function is reduced in the diabetic state as well as to progress in new research and development that will be useful in medical care and drug discovery.

In this paper, we described the insulin control network of both the neural stem cells in the brain and the endocrine cells in the pancreas based on their involvement with diabetes patients, and the accumulating academic information linked to overcoming the diminished functions of stem cells whose functions have deteriorated will be very useful. In the future, it appears that it might also be possible to contribute to elucidating the pathology not only of diabetes but also of neuropsychiatric diseases whose risk increases as the pathology of diabetes progresses or to the search for new therapeutic reagents for the treatment of human neuropsychiatric diseases and to the development of treatment techniques.
